# Who pays and who benefits? How different models of shared responsibilities between formal and informal carers influence projections of costs of dementia management

**DOI:** 10.1186/1471-2458-11-793

**Published:** 2011-10-12

**Authors:** Victor Vickland, Joel Werner, Thomas Morris, Geoff McDonnell, Brian Draper, Lee-Fay Low, Henry Brodaty

**Affiliations:** 1 Dementia Collaborative Research Centre, School of Psychiatry, University of New South Wales, Sydney, Australia; 2 Centre for Health Informatics, Australian Institute of Health Innovation, University of New South Wales, Sydney, Australia; 3 Academic Department for Old Age Psychiatry, Prince of Wales Hospital, Sydney, Australia

## Abstract

**Background:**

The few studies that have attempted to estimate the future cost of caring for people with dementia in Australia are typically based on total prevalence and the cost per patient over the average duration of illness. However, costs associated with dementia care also vary according to the length of the disease, severity of symptoms and type of care provided. This study aimed to determine more accurately the future costs of dementia management by taking these factors into consideration.

**Methods:**

The current study estimated the prevalence of dementia in Australia (2010-2040). Data from a variety of sources was recalculated to distribute this prevalence according to the location (home/institution), care requirements (informal/formal), and dementia severity. The cost of care was attributed to redistributed prevalences and used in prediction of future costs of dementia.

**Results:**

Our computer modeling indicates that the ratio between the prevalence of people with mild/moderate/severe dementia will change over the three decades from 2010 to 2040 from 50/30/20 to 44/32/24.

Taking into account the severity of symptoms, location of care and cost of care per hour, the current study estimates that the informal cost of care in 2010 is AU$3.2 billion and formal care at AU$5.0 billion per annum. By 2040 informal care is estimated to cost AU$11.6 billion and formal care $AU16.7 billion per annum. Interventions to slow disease progression will result in relative savings of 5% (AU$1.5 billion) per annum and interventions to delay disease onset will result in relative savings of 14% (AU$4 billion) of the cost per annum.

With no intervention, the projected combined annual cost of formal and informal care for a person with dementia in 2040 will be around AU$38,000 (in 2010 dollars). An intervention to delay progression by 2 years will see this reduced to AU$35,000.

**Conclusions:**

These findings highlight the need to account for more than total prevalence when estimating the costs of dementia care. While the absolute values of cost of care estimates are subject to the validity and reliability of currently available data, dynamic systems modeling allows for future trends to be estimated.

## Background

The type of care provided in Australia to persons with dementia is primarily dependent on the severity of the disease. In the milder stages of dementia, care is typically at home and provided by a relative (informal carer). However, as severity increases, informal care is often supplemented by professional carers (formal care) largely funded through the Commonwealth Aged Care Program that target both the person with dementia and their carer e.g. Community Aged Care Packages, Extended Aged Care At Home Dementia Packages, and Dementia Respite care. In the more severe stages of the disease, persons with dementia are primarily institutionalised in residential aged care facilities that also receive considerable Commonwealth funding. Access to formal community and residential care in Australia requires an assessment by an Aged Care Assessment team and formal approval that the individual has sufficient disability and needs to warrant Commonwealth funding [1]. This progression incurs a significant cost to both the individual and the community, which will increase as the prevalence of dementia increases [2].

According to a recent review [3], the few studies that have attempted to model the future costs of caring for people with dementia in Australia have assumed that total cost will increase relative to total disease prevalence, and that interventions designed to delay disease onset and/or progression will reduce prevalence, and thereby cost.

Such assumptions limit the accuracy of cost estimates unless they account for costs associated with patient location, the severity of dementia, and the type of care (informal/formal). Quentin et al. [4] recently published a comprehensive review that highlighted the need to account for the influence of these variables when estimating the cost of illness in studies of dementia. They found that cost of care doubles as dementia severity progresses from the mild to severe stage, and that patient location determines the relative proportion of informal/formal care a patient receives. Such results confirm the importance of a multidimensional approach to the calculation of total costs of care for people with dementia.

The accuracy of cost estimates can be further enhanced by accounting for projected dementia prevalence and the impact of interventions on dementia severity-specific prevalence. Dementia prevalence in Australia will continue to increase to 2040 and beyond [2]. The relative distribution of dementia severity-specific prevalence will also change over time [2]. These factors influence cost estimates. As the severity of dementia increases, so does the need for informal and formal care, but once people with dementia are placed in institutional care most of the costs are relatively fixed, irrespective of cognitive decline or activities of daily living (ADL) [5].

Numerous studies, such as those of Nepal and colleagues [3], Quentin and colleagues [4], and the Alzheimer's Association [6] have varied in their estimates of predicted costs associated with future projections of dementia prevalence, but all demonstrate predicted increases in both the prevalence and related cost of dementia. These projections do not, however, attempt to estimate the future cost of dementia care by taking into account the influence of location, type of care, and dementia severity over time. This study aimed to build upon such models by more accurately determining the costs associated with dementia by taking these factors into account.

We developed a computational model to forecast the prevalence of mild, moderate and severe dementia in Australia 2010-2040 [2]. Data from a variety of sources [2][7-9] were recalculated to redistribute total prevalence according to the location, care type required, and dementia severity of the person with dementia. Cost data from an alternative set of sources [7][10,11] were applied to our redistributed prevalence estimates, thereby providing a unique estimate of dementia care costs in Australia 2010-2040.

Virtual experiments examined the impact on total prevalence of delaying disease progression by 2 years and delaying disease onset by 2 years. Cost estimations were carried out for each of these scenarios.

In essence, predictive modeling relies on sourcing the most valid and reliable data available, and then recalculating those data over time. As such a process relies on various estimates and assumptions, computer modeling in research does not aim to provide, nor can it report, definitive results. Rather, predictive modeling attempts to demonstrate possible outcomes derived from the parameters included, be they theoretical or actual.

This study aggregated data from a variety of sources to produce current (2010) estimates of dementia prevalence by location, care type, dementia severity, and relative care costs. Prevalence was then extrapolated across the next three decades using the dementia prevalence model [2], and costs recalculated accordingly.

Taking 2010 values as a baseline measure of prevalence and cost, it is the relative changes predicted by the model over time that are of most interest - absolute values are (due to the limitations of available data) a "best guess", and easily recalculated if/when more accurate data becomes available. However, the direction and magnitude of prevalence shift over time is considerably more accurate.

As such, the main focus of the current study is the dynamism of prevalence shifts over time, and the consequent impact on total cost of care.

### Aims

1. To integrate research estimates of the hours of informal and formal care provided to dementia patients according to the severity of disease and patient location/care setting.

2. To project the severity-specific prevalence of dementia in Australia 2010 - 2040 using the dementia prevalence model [2].

3. To examine impact of an intervention to slow disease progression by 2 years.

4. To examine impact of an intervention to delay disease onset by 2 years.

5. To estimate the change in costs of informal and formal care for dementia patients in Australia 2010 - 2040.

## Methods

The dementia prevalence model was used to calculate severity-specific prevalences of dementia (Australia 2010 - 2040) as well as the impact over time of interventions introduced in 2010 (Table 1)[2]. The Dementia Prevalence Model is a computer model designed to estimate the prevalence of dementia in Australia, and involves ascribing characteristics representative of dementia patients (such as age, mortality, dementia severity), and then 'aging' these over 30 years. The model identified various trajectories of dementia prevalence depending on whether interventions were established and at what stage these were introduced. As is common in the literature [3][8][12], we chose to model feasible interventions that could either a) delay the disease progression from mild dementia to moderate dementia by 2 years (i.e. everyone with mild dementia would take an additional two years to progress to a more moderate form of the disease), or b) delay the onset of dementia by 2 years (i.e. everyone would take an additional 2 years to get dementia, but then progress to a more moderate form of the disease at the same rate as if there had been no intervention). While such interventions are not currently available, advances in the diagnosis of dementia and dementia treatment will likely play a role in delaying the onset or slowing the progression sometime in the near future. For example, the existence of a healthy lifestyle might be a preventative measure in developing dementia [13], and advances in the early detection of dementia might delay onset by allowing earlier pharmacological treatment.

**Table 1:** Dementia prevalence in Australia 2010 - 2040 with and without interventions

	No intervention				Delay progression				Delay onset			
	**Mild**	**Mod**	**Severe**	**Total**	**Mild**	**Mod**	**Severe**	**Total**	**Mild**	**Mod**	**Severe**	**Total**

**2010**	117,000	71,000	44,000	232,000	117,000	71,000	44,000	232,000	117,000	71,000	44,000	232,000

**2020**	168,000	111,000	75,000	354,000	204,000	96,000	64,000	364,000	139,000	57,000	94,000	290,000

**2030**	242,000	166,000	120,000	528,000	295,000	144,000	104,000	543,000	202,000	84,000	140,000	426,000

**2040**	327,000	235,000	182,000	744,000	402,000	205,000	159,000	766,000	279,000	120,000	210,000	609,000

The data provided by the dementia prevalence model [2], the work of Brookmeyer and colleagues [8], and reports concerning dementia prevalence and cost by Access Economics [7][9] were used to calculate the relative prevalence of dementia severity (mild/moderate/severe) by location and care type. Total dementia prevalence for Australia in 2010 was distributed between dementia severities by location and care type (see Table 2: 2010 values).

**Table 2:** Dementia severity by location and type of care

Total dementia population (2010)					
N = 232000			Severity	Percent of total	Prevalence (2010)
			
Location		Type of care setting			
			Mild (0.6)	21%	48720
			
Home (35%)		Community; informal care with no formal care	Moderate (0.3)	10.50%	24360
			
			Severe (0.1)	3.50%	8120

			Mild (0.6)	15%	34800
			
Home (25%)		Community; mixture of informal and formal care	Moderate (0.3)	7.50%	17400
			
			Severe (0.1)	2.50%	5800

Institutions (40%)			Mild (0.35)	14%	32480
		
RAC 1-4	RAC 5-8	Institutions; mixture of informal and formal care	Moderate (0.3)	12%	27840
			
(33%)	(7%)		Severe (0.35)	14%	32480

			Total	100%	232000

These models [2][8] provided the ratio of mild:moderate:severe dementia (5:3:2 for 2010) and Access Economics [7] provided percentage splits for location and type of care. From these data sets values for dementia severity by location/care type were triangulated.

Costs specific to the dependent relationship between these variables (location, care type, dementia severity) were then attributed to the dementia population 2010-2040 using 2010 Australian dollar (AU$) values; (approximately parity with US$ in Sept 2011). Values for informal/formal hours of care per person with dementia, and the cost of informal/formal care per hour per person with dementia were calculated from the data of Access Economic [7][9], the World Health Organisation [10] and the Australian Institute of Health and Welfare [11].

For formal hours of care, values ranging from 39 hr/pa for a person with mild dementia living at home with a family, to 1512 hr/pa for a person with severe dementia living in a residential aged care facility were adopted [[7] Table E1]. The assumptions regarding informal hours of care are not clearly reported in published literature. Total hours of care per week for a person living with dementia at home are estimated to be about 8.5 hr for mild, 25.0 hours for moderate and 41.5 hours for severe dementia [[9] Table 15 p.47]. Total hours per annum can be calculated for informal care as 445 (mild), 1304 (moderate), and 2165 (severe). Similarly, if care at home is shared with help from formal carers the informal hours of care are reduced to 404 (mild), 619 (moderate) and 1141 (severe) hours per annum. Informal care hours when the patient was living in residential aged care (distinguished from social visits) were at the level of only 1 hr (mild), 2.5 hr (moderate) and 5 hr (severe) per week [9]. Hours of care for patients grouped by location, type of care received, and by severity of dementia are presented in Table 3. The literature is unclear as to how reliable these estimates are. Estimates of the the hourly cost of care are AU$27 (2009 values) for low level (informal) care [[7] p.49] with an additional increase by 20% for high level (formal) [11] care to AU$33.

**Table 3:** Hours of informal/formal care per person with dementia per year by dementia severity, location, and type of care (estimates from available literature)

Total dementia population (2010)					
N = 232000			Severity	Hours per person with dementia per year	
			
Location		Type of care setting		Informal	Formal
			Mild (0.6)	445	.
			
Home (35%)		Community; informal care with no formal care	Moderate (0.3)	1304	.
			
			Severe (0.1)	2165	.

			Mild (0.6)	404	39
			
Home (25%)		Community; mixture of informal and formal care	Moderate (0.3)	619	685
			
			Severe (0.1)	1141	1023

Institutions (40%)			Mild (0.35)	75	1355
		
RAC 1-4	RAC 5-8	Institutions; mixture of informal and formal care	Moderate (0.3)	150	1512
			
(33%)	(7%)		Severe (0.35)	250	1512

Costs were calculated according to the formula (where s - severity, and l - location):

$$\text{Cos}{\text{t}}_{\left(\text{Total}\right)}=\sum_{\left(\text{s},\;\text{l}\right)}\text{Cos}{\text{t}}_{\text{Informal}\;\text{care}}+\sum_{\left(\text{s},\;\text{l}\right)}\text{Cos}{\text{t}}_{\left(\text{Formal}\;\text{care}\right)}$$

Costs were calculated by multiplying each of the location/care-type/dementia severity specific prevalence values by the number of informal/formal care hours per person with dementia (pwd) per year to arrive at the total hours of care per pwd per year. These values were then multiplied by the care cost per hour (2010 equivalent).

For example;

• In 2010, 5800 patients with severe dementia live at home and receive both informal and formal care.

• These patients each receive 1141/1023 hours of informal/formal care respectively per year.

• This translates to 7.0 million hours of informal care and 5.9 million hours of formal care provided to these patients in 2010.

• At a care cost of $27/hour, this equates to AU$188 million for informal care and AU$160 million for formal care for persons with severe dementia who live at home (2010).

The impact of interventions to delay disease progression/onset on dementia prevalence was then calculated.

For further information on the Dementia Prevalence Model, including a sensitivity analysis, see [2][14].

## Results

**Figure 1:**
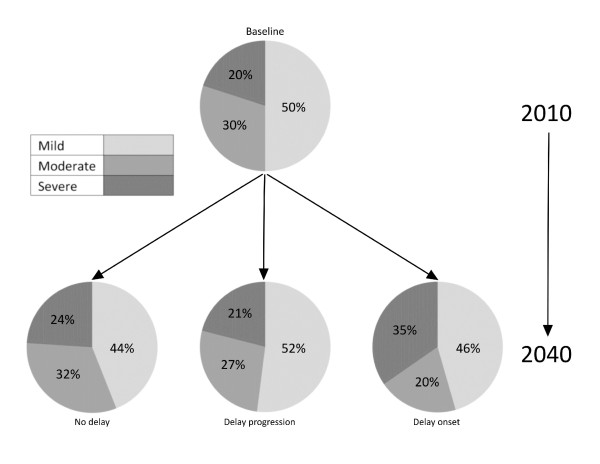
Relative change in severity-specific dementia prevalence in Australia 2010-2040 with and without interventions

An examination of the severity specific prevalence of dementia in Australia (2010 - 2040) reveals that severity ratios change over time (Figure 1). Even in the absence of an intervention (no delay in onset/progression), severity ratios will change due to changing demographics [2].

Without the influence of an intervention there will be 6% fewer cases of mild dementia by 2040. The introduction of interventions to delay disease progression/onset by 2 years likewise alters the severity-specific prevalence of dementia. If all persons with mild dementia take an extra two years to progress to moderate dementia, there will be 8% more cases of mild dementia in 2040 relative to no delay. If an intervention introduced in 2010 delays disease onset by 2 years, there will only be 2% more cases of mild dementia in 2040 relative to no delay. There will, however, be 11% more cases of severe dementia due to the accumulating effect of already diagnosed cases.

Such dynamic interactions impact on total cost of care over time. Informal/formal costs of care in 2010 for patients grouped by location and type of care received, and by severity of dementia are presented in Table 4 (2010 values). Projected costs of dementia in Australia 2010 - 2040 are presented in Table 5.

**Table 4:** Informal/formal costs by location and type of care, and dementia severity for 2010

Total dementia population (2010)					
N = 232000			Severity	Informal (AU$)	Formal (AU$)
			
Location		Type of care setting			
			Mild	585,370,800	.
			
Home (35%)		Community; informal care with no formal care	Moderate	857,666,000	.
			
			Severe	474,654,600	.

			Mild	385,236,000	37,584,000
			
Home (25%)		Community; mixture of informal and formal care	Moderate	328,860,000	321,813,000
			
			Severe	187,920,000	160,201,800

Institutions (40%)			Mild	65,772,000	1,452,343,200
			
RAC 1-4	RAC 5-8	Institutions; mixture of informal and formal care	Moderate	112,752,000	1,378,080,000
		
(33%)	(7%)		Severe	219,240,000	1,714,944,000

			Total	3,217,472,200	5,064,966,000

**Table 5:** Cost of dementia care (informal/formal) in Australia 2010 - 2040 with and without interventions ($A billion dollars 2010 equivalent)

	No intervention			Delay progression (2 yr)			Delay onset (2 yr)		
	**Informal**	**Formal**	**Total**	**Informal**	**Formal**	**Total**	**Informal**	**Formal**	**Total**

**2010**	3.2	5.0	8.2	.	.	.	.	.	.

**2020**	5.1	7.8	12.9	4.5	7.8	12.3	4.8	6.5	11.3

**2030**	8.0	11.7	19.7	7.1	11.7	18.7	7.2	9.6	16.8

**2040**	11.6	16.7	28.3	10.2	16.6	26.8	10.5	13.8	24.3

In the absence of intervention, the total cost of dementia care in Australia in 2010 is AU$8.2 billion dollars, 61% of which is accounted for by formal care costs. In 2040 the total cost will rise to AU$28.3 billion dollars, 59% of which will be accounted for by formal care costs (Table 5). An intervention to slow the progression of dementia by 2 years will result in a 5% saving (AU$1.5 billion) per annum by 2040; an intervention to delay the onset of dementia by 2 years will result in a 14% saving (AU$4 billion) per annum by 2040.

**Figure 2:**
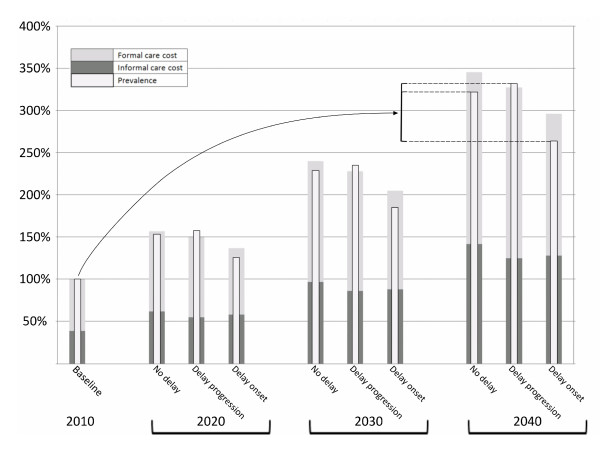
**Relative change in dementia prevalence and formal/informal costs in Australia 2010-2040 with and without interventions**. Dotted lines illustrate the difference in prevalence between the scenarios at 2040.

The relationship between cost and prevalence is dynamic, and mediated by the interaction between dementia severity and formal/informal costs. Figure 2 presents the dynamic relationship between cost and prevalence over time. Such dynamic relationships are consistent beyond absolute cost estimates, which fluctuate according to the accuracy/reliability of available data.

Relative to a 2010 baseline (100%), 2040 prevalence has increased to 322% in the no delay scenario. Under the influence of an intervention to delay progression by 2 years, there is a 10% relative increase in 2040 prevalence, taking it to 332%. On the contrary, an intervention to delay onset by 2 years will result in a 58% relative reduction of prevalence, to 264% of 2010 values. This is the comparison highlighted on the graph. The same logic can equally be applied to informal, formal, or total cost.

The delay onset scenario results in the lowest overall values for both total cost and prevalence, but the highest cost per person with dementia (Table 6). Formal costs per person with dementia are relatively stable across all scenarios - it is the informal care cost that is changing most.

**Table 6:** Cost of dementia care per person with dementia per annum (informal/formal/total) in Australia 2040 with and without interventions ($A)

	No Intervention	Delay Progression (2 yr)	Delay Onset (2 yr)
**Informal**	15,600	13,400	17,200

**Formal**	22,400	21,600	22,600

**Total**	38,000	35,000	40,000

As these results clearly demonstrate, estimating the care costs of dementia based on total prevalence alone ignores many influential dynamics:

• Prevalence and cost of care both increase over time

• In the absence of any intervention (no delay) by 2040;

○ Total prevalence increases to 322% relative to 2010 values (100%)

○ Total formal costs increase to 203% relative to 2010 values (100%)

○ Total informal costs increase to 142% relative to 2010 values (100%)

• An intervention to delay disease progression by 2 years will, over time;

○ Increase total prevalence relative to the no delay condition

○ Reduce overall cost relative to the no delay condition

• An intervention to delay disease onset by 2 years will, over time;

○ Reduce total prevalence relative to the no delay condition

○ Reduce overall cost relative to the no delay condition

The current analysis does not take into account the cost of interventions as it is difficult to estimate these costs when such treatments are not yet viable. Possible interventions could take any form (e.g. regular exercise or pharmacological treatments) which would vary considerably in their respective cost. Given this ambiguity, attempting to estimate the cost of these potential interventions would be inappropriate. In order to demonstrate the potential cost of a hypothetical intervention, the current per annum cost of donepezil [15] was used as representing the possible cost of a treatment that would either delay progression or onset of dementia (Table 7).

**Table 7:** Cost of hypothetical intervention in Australia 2010 - 2040 ($A dollars 2011 equivalent)

	Delay Progression	Delay Onset
**2010**	237,042,000	237,042,000

**2020**	413,304,000	281,614,000

**2030**	597,670,000	409,252,000

**2040**	814,452,000	565,254,000

Values obtained by multiplying the current per annum cost of donepezil (~AU$2026 per person) by the projected prevalence of mild dementia in 2010-2040. While an intervention to delay onset would be administered before the onset of mild dementia, costs were calculated on this basis in the absence of appropriate data

## Discussion

The current study addresses a gap in the literature that evaluates the cost of dementia [3]. The formal and informal costs of care in different stages of dementia progression were estimated for Australia 2010-2040. The fiscal impact of therapeutic interventions to delay disease onset and progression was similarly evaluated. Previous studies have not simultaneously accounted for severity specific costs while recognising the distinction between formal and informal costs of care. The current cost of dementia in Australia is calculated to be AU$8.2 billion per annum, with formal care accounting for 61% of the total. By 2040, these costs will have increased to AU$28.3 billion per annum, 59% of which would be spent on formal care. As noted previously, absolute values such as these need to be interpreted with caution due to the lack and inconsistency of data available. However, estimates of the prevalence shift over time are considerably more accurate, and are more likely to remain constant with changes in these values. Our results indicate that slowing the progression of dementia by 2 years will result in a 10% relative increase in the total prevalence of dementia by 2040 relative to the no delay scenario. Such a result, while not intuitive, is due to higher rates of severe dementia in the no delay scenario, and the respective increase in mortality. Despite this increased prevalence, the relative cost of care will be reduced by AU$1.5 billion per annum, as the milder stages of dementia are associated with lower costs. Delaying the onset of dementia by 2 years will result in a relative reduction of both prevalence and total cost. These results highlight the need for cost estimation to account for more than total prevalence.

A more interesting dynamic is observed when the annual cost per person with dementia is examined. Under the influence of an intervention that successfully delays the onset of dementia by 2 years both prevalence and total cost are reduced relative to no intervention. However, the total cost of care per person with dementia per annum in 2040 is $2000 more expensive than the no delay condition, and $5000 more expensive than the delay progression condition.

In light of these findings, it is pertinent to question traditional notions of success when evaluating potential interventions - be they economic or clinical in nature. The intervention most beneficial at a population level (delay onset; lowest prevalence/total cost) places the greatest fiscal burden on individuals and their families. Whereas the intervention that leaves society with the greatest number of people with dementia also results in the lowest cost per person with dementia per annum. These differences in cost per person are driven by informal costs, and would thereby be more likely a burden on the individual than the government. In designing future objectives, policy decision makers need to question who pays, and who benefits from any potential interventions.

The driving force behind these dynamics is severity-specific prevalence. The relative number of patients with mild/moderate/severe symptoms changes over time, and differentially according to the influence of interventions. Disease progression is strongly associated with a transition from mostly informal to mostly formal care. Different types of care and different care settings are associated with different costs.

Many factors determine the current costs, but there is agreement that the main component is the cost of formal care, which rises as dementia progresses. People with moderate dementia often transition from mostly informal to mostly formal care. This includes the shift from home based care in the community to residential or institution-based care. Benefits of care interventions, medications and non-medication therapies, policies of early detection and policies of providing more formal care in the community are justified by how much they avoid institutionalisation. Controversies arise because there are large differences in informal care hours and costs. In addition, the effects of dementia on the quality of life of both people with dementia and their carers are difficult to quantify with appropriate economic social and environmental measures (triple line accounting) that include distributions of costs and benefits among individuals.

For a clearer approach to costing we recommend the following:

• Consistent definitions of progression of dementia using and reporting multiple dimensions, the level of cognitive impairment, estimated time from onset of symptoms, level of assistance with activities of daily living (ADL) or disability weights, with an agreed comorbidity scale for both physical and mental disorders.

• Clearly distinguish between cost of illness and cost of caring for people with dementia. In most cases it is better to count the whole cost of individual care rather than artificially split this cost in complex patients into fractional disease components among many interacting diagnoses.

• Focus on costing the transition from informal to formal care within the moderate stage of dementia. This includes using the person with disability (with their network of informal and formal carers) as the unit of analysis of costs and including the value of positive experiences and wellbeing as benefits to participants in this care network [16].

While it is likely that similar trajectories of prevalence exist in other western countries, where rates of dementia and the types of care are comparable, estimates of dementia prevalence can vary considerably. For example, the current model differs to a report by the Alzheimer's Association [6] in estimates of baseline and future rates of mild, moderate and severe dementia with or without intervention. Specifically the report demonstrates higher rates of severe dementia and lower rates of mild dementia than the current study (e.g. 28:31:41 [6] compared to 50:30:20 (mild:moderate:severe) at 2010 baseline). Similar levels of variability can be seen in the projected costs of formal and informal care of dementia in Australia. A recent assessment of future costs [11] estimated the cost of dementia in 2030-2031 at AU$4.5 billion, considerably less than the AU$19.7 billion projected by this study. A possible explanation for such a finding is that the current study accounted for not only formal costs of care, as the other estimate was based, but also costs associated with informal care. It is likely that this discrepancy is also due to more recent estimates of dementia prevalence being utilised in the current study. The capability of any model to adequately account for prevalence rates and costs associated with dementia is largely reliant on the data available to this end, and is modulated by social norms, expectations and the provision of care in these varying populations. As a result, comparing studies is not always appropriate as each study aims to achieve a 'best guess' in their estimates and assumptions. Applying the dementia prevalence model with a variety of parameters in various populations would be a useful indicator of the cost of formal and informal care in Australia and internationally.

The current study is limited by the costing data currently available to model, and perhaps this goes some way to explaining the lack of research in the area [3]. An accurate estimation of formal and informal costs by severity is difficult at present. Available published data are fragmented and different government agencies have different reporting requirements, with generally only the global costs of formal and informal care available for analysis. Such inconsistency disallowed many costs to be included in this study.

A more collaborative approach to this problem will enhance cost estimates and future policy decisions. Assumptions regarding duration of illness [17] and average life expectancy [18] are limited to current state of knowledge in these areas. For example, if delay in onset of dementia increases life span, this would merely shift costs some years hence rather than reduce them. In future these assumptions may change significantly and so will the projected outcomes. As patient and carer networks change frequently, especially during the moderate phase of dementia, methods that account for this dynamic dimension will be able to allocate costs and benefits using activity based costing methods. Costs can be allocated to both disease states and transitions, independent of states. For example the costs associated with newly diagnosed dementia cases might occur at any stage of dementia, rather than these costs always allocated to the mild stage of dementia. New advances with biomarker technology for early detection of dementia may in fact increase demand on services in very early stages of disease [19].

Many additional factors influence the costs of care, and these may change in future. For example, labour costs may change relative to a supply-demand cycle for workers in residential aged care facilities. The replacement costs for informal care may also be calculated differently if the retirement age increases. Carers 65 years and older and still in the work force will convert from paid employment to a role of informal carer at much higher replacement cost.

A further limitation of this study is that the costs associated with the interventions could not be included in the analysis as the cost of such interventions are yet to be known. It is obvious that costs will be incurred with any measure introduced to delay or slow the progression of dementia. While the cost of a hypothetical intervention was estimated based on the current cost of donepezil, costs of potential treatments remain unknown.

Estimating care costs for dementia, now and in the future, requires a more detailed analysis than total prevalence alone can provide. The potential of interventions that prolong disease progression, or delay disease onset to produce savings in terms of prevalence, or cost of care, or both is exciting. However, savings at an aggregated level are not necessarily passed on to the individual. The complexities of predicting trends in such a dynamic system necessitate that clinicians and policy makers take time to consider who pays, and who benefits from interventions.

## Conclusions

The few studies that have attempted to model the future costs of caring for people with dementia in Australia have assumed that total cost will increase relative to total disease prevalence, and that interventions designed to delay disease onset and/or progression will reduce prevalence, and thereby cost. Such assumptions limit the accuracy of cost estimates unless they account for costs associated with patient location, the severity of dementia, and the type of care (informal/formal).

Results from the dementia prevalence model predict that both prevalence and cost of care increase over time, however, an intervention to delay disease progression by 2 years will increase prevalence and reduce overall care costs over time. On the other hand, an intervention to delay disease onset by 2 years will reduce both prevalence and overall care costs over time.

These findings highlight the need to account for more than total prevalence when estimating the costs of dementia care. Variables such as location, care type, and dementia severity can significantly enhance the accuracy of estimates, especially when predicting the future costs of dementia care. While the absolute values of cost of care estimates are subject to the validity and reliability of currently available data, dynamic systems modeling allows for future trends to be estimated.

## Competing interests

The authors declare that they have no competing interests.

## Authors' contributions

VV conceptualized the study design, carried out virtual experiments and drafted the paper. JW performed statistical analysis and graphical presentation of the results and drafted the paper. TM contributed to graphical presentation of the results, addressed comments from reviewers and prepared manuscript for publication. GMD provided cost analysis. BD, LFL and HB contributed to the analysis and interpretation of results and reviewed drafts of the manuscript. All authors read and approved the final manuscript.

## Pre-publication history

The pre-publication history for this paper can be accessed here:

http://www.biomedcentral.com/1471-2458/11/793/prepub
